# Phosphorus Nutrient Management through Synchronization of Application Methods and Rates in Wheat and Maize Crops

**DOI:** 10.3390/plants9101389

**Published:** 2020-10-19

**Authors:** Rafiullah, Muhammad Jamal Khan, Dost Muhammad, Shah Fahad, Muhammad Adnan, Fazli Wahid, Saud Alamri, Farmanullah Khan, Khadim Muhammad Dawar, Inam Irshad, Subhan Danish, Muhammad Arif, Amanullah, Shah Saud, Bushra Khan, Ishaq Ahmad Mian, Rahul Datta, Tayebeh Zarei, Anis Ali Shah, Musarrat Ramzan, Muhammad Zafar-ul-Hye, Maria Mussarat, Manzer H. Siddiqui

**Affiliations:** 1 Department of Agriculture, University of Swabi, Swabi 23561, Pakistan; rafiullah@uoswabi.edu.pk (R.); madnan@uoswabi.edu.pk (M.A.); fazliwahid@uoswabi.edu.pk (F.W.); 2 Department of Soil and Environmental Sciences, the University of Agriculture Peshawar, Peshawar 25000, Pakistan; Jamal@aup.edu.pk (M.J.K.); dostms76@gmail.com (D.M.); khadimdawar@yahoo.com (K.M.D.); ishaqmian@aup.edu.pk (I.A.M.); drmaria@aup.edu.pk (M.M.); 3 Hainan Key Laboratory for Sustainable Utilization of Tropical Bioresource, College of Tropical Crops, Hainan University, Haikou 570228, China; 4 Department of Agronomy, The University of Haripur, Khyber Pakhtunkhwa 22620, Pakistan; 5 Department of Botany and Microbiology, College of Science, King Saud University, Riyadh 2455, Saudi Arabia; saualamri@ksu.edu.sa (S.A.); mhsiddiqui@ksu.edu.sa (M.H.S.); 6 Soil and Water Conservation, Directorate of Soil and Water Conservation, Peshawar, Khyber Pakhtunkhwa 25120, Pakistan; sd9685075@yahoo.com; 7 Institute of Soil and Environmental Sciences, University of Agriculture Faisalabad, Punjab 38000, Pakistan; Inamirshad12@gmail.com; 8 Department of Soil Science, Faculty of Agricultural Sciences and Technology, Bahauddin Zakariya University, Multan, Punjab 60800, Pakistan; zafarulhye@bzu.edu.pk; 9 Department of Agronomy, The University of Agriculture, Peshawar 25130, Pakistan; marifkhan75@aup.edu.pk (M.A.); amanullah@aup.edu.pk (A.); 10 Department of Horticulture, Northeast Agriculture University, Harbin 150036, China; saudhort@gmail.com; 11 Department of Environmental Sciences, University of Peshawar, Peshawar 25120, Pakistan; bushraasu@uop.edu.pk; 12 Department of Geology and Pedology, Faculty of Forestry and Wood Technology, Mendel University in Brno, Zemedelska1, 61300 Brno, Czech Republic; 13 Department of Agronomy and Plant Breeding, Yasouj University, Yasouj 7591874934, Iran; t.zarei@stu.yu.ac.ir; 14 Department of Botany University of Narowal, Punjab 51801, Pakistan; anisalibot@gmail.com; 15 Department of Botany, The Islamia University of Bahawalpur, Punjab 63100, Pakistan; musarrat.ramzan@iub.edu.pk

**Keywords:** band application, foliar application, grain yield, nitrogen, phosphorus, calcareous soil

## Abstract

Management of inorganic fertilizer is very important to obtain maximum crop yield and improved nutrient use efficiency in cereal crops. Fixation of phosphatic fertilizers in alkaline soils due to calcareousness is one of the major hurdles. It induces phosphorus nutritional stress that can decrease the yield of maize and wheat. Selection of a suitable application method and proper stage of crop for phosphorus (P) fertilizer has prime importance in better uptake of P and crop production. Among different application methods, soil and foliar application are widely adopted. In wheat and maize, knee height + tasseling and stem elongation + booting are critical stages towards P deficiency. That is why field trials were conducted to evaluate the supplemental effect of foliar P on maize and wheat yields. For that, 144 mM KH_2_PO_4_ was applied as foliar at knee height + tasseling and stem elongation + boot stages in maize and wheat, respectively. Soil application of 0, 20, 40 and 60 kg P ha^−1^ was done through broadcast and band methods. Results showed that foliar spray of 144 mM KH_2_PO_4_ at knee height + tasseling and stem elongation + boot stages in wheat and maize significantly enhanced grains yield and phosphorus use efficiency (PUE) where P was applied as banding or broadcast at the time of sowing. A significant decreasing trend in response to increasing soil P levels validated the efficacious role and suitability of foliar P. In conclusion, the use of P as foliar at knee height + tasseling and stem elongation + boot stages is an efficacious way to manage P fertilizer.

## 1. Introduction

Phosphorus is an essential element for animals and plants. Its long term management is very important for ending hunger of the world. In global food security, management of phosphorus is also listed by the United Nations’ Sustainable Development Goals (SDGs) [1]. The inadequacy of phosphorus (P) is a worldwide concern that is not limited to Western industrialized nations. Until now, many countries of the world have been highly dependent on importing mineral P fertilizers for agriculture [2]. It is an important macronutrient for optimum growth, metabolism and development of crops [3]. Worldwide, P is considered a key factor for increasing yield, after nitrogen [4,5]. It has higher requirements in most cereal and vegetal crops [6,7]. Phosphorus is found as primary and secondary ortho-phosphate (mineralized forms). The high rate of adsorption on clay surfaces usually causes fixation of primary and secondary ortho-phosphate in soil [8]. It also become precipitated with Ca^+2^ and Mg^+2^ under alkaline pH or with Fe^+2^ and Al^+3^ at acidic pH [3]. Therefore, the available P in cultivatable soil become as low as 0.1 mg kg^−1^. More than 30–40% of the agricultural lands across the world are deficient in P [9]. In such soils, P is regulated through exogenous addition of fertilizers to meet P requirement in high yielding crops [10]. Out of the total, only 20% of P fertilizer is used by the crops while the remaining 80% is lost via fixation, leaching, etc., resulting in less P use efficiency [11]. Furthermore, the use of organic fertilizers leads to the risk of xenobiotic contamination [12,13,14,15].

To overcome this problem, organic amendments, bio-fertilizers and foliar fertilization are widely used practices that can increase phosphorus uptake in plants [16,17,18,19,20,21,22]. Among the different techniques, foliar fertilization has better potential to correct nutritional deficiencies in plants caused by the improper supply of nutrients to roots [23,24,25]. This practice is usually more economical and effective under certain conditions [26] and is generally considered efficient to supply nutrients quickly to a target organ. This technique is particularly adapted and important for crops to achieve maximum and best yield when crop nutrient demand is not completely fulfilled during the crop growth period. The foliar phosphorus could augment the soil-applied phosphorus that may increase the phosphorus use efficiency and reduce the crop dependence on soil phosphorus. Resultantly, it will help in reducing the recommended levels of phosphorus if applied in conjunction with foliar phosphorus [27].

Silberstein and Wittwer [28] and Dixon [29] suggested that foliar application of phosphorus could be the most effective way for a grower to supply phosphorus in late stages of the crop and increase fertilizer use efficiency; however, this may vary with crop type, soil and climatic conditions. Halo [27] observed that the initial symptoms of phosphorus deficiency that appeared in wheat within 20–25 days of sowing were corrected by spraying ammonium phosphate. In another study, Ali et al. [30] reported that foliar phosphorus application was more responsive in phosphorus-deficient conditions than phosphorus-sufficient soils. Benbella and Paulsen [31] showed that foliar P application at anthesis with 5 to 10 kg KH_2_PO_4_ ha^−1^ (1.1 to 2.2 kg P ha^−1^) enhanced wheat grain yield up to 1.0 Mg ha^−1^.

Numerous factors influence the efficacy of foliar-applied fertilizers and may elucidate the often-reported discrepancy in plant response to foliar nutrition. Foliar application of phosphorus enhances its use efficiency [32], which could reach even up to 100% when 4 kg P ha^−1^ was applied [33]. The most important factors in foliar application are a suitable dose, source and time of application, as well as the number of sprays to be applied. Arif et al. [34] reported higher wheat yield with phosphorus solution sprayed at the tillering, jointing and booting stages of the crop. Similarly, Mosali et al. [33] reported maximum benefit from spraying 2 kg P ha^−1^ at the anthesis stage of wheat as compared to 1 or 4 kg P ha^−1^ and at other stages of crop growth.

That is why, keeping in mind the importance of P application methods for P management in cereal crops, the current study was conducted with the aim to synchronize the method of P application in wheat and maize cropping systems. To find whether soil-applied P with the combination of foliar-applied P increases crop growth and yield or not. It is hypothesized that P use efficiency may be improved in wheat and maize through synchronization of P application method in cereal crops.

## 2. Results

### 2.1. Plant Height

Maize and wheat plant heights were significantly increased with increase in P levels and foliar application of 144 mM KH_2_PO_4_ solution irrespective of soil application methods (Table 1). When averaged across the application methods and years, the plant height of maize increased from 197 cm in control to 216 cm with 60 kg P ha ^−1^ without foliar application of P, whereas when the soil application of P was supplemented with foliar application, the increase in plant height varied from 205 cm in control (with soil P) to 221 cm where a high rate of soil P was applied. When averaged across the soil-applied P treatment, there were significant variations in plant height among the plot depending on whether it was sprayed or not sprayed. The same trend was observed for wheat and the plant height obtained at 20 kg P ha^−1^ with foliar 144 mM KH_2_PO_4_ solution which was better (96 cm) than the plant height obtained with 40 kg P ha^−1^ without foliar P when values were averaged across the years and soil application methods (Table 1). These results revealed a decrease in dependence on soil-applied P and saving of fertilizer due to foliar P application. By comparing the fertilizer application methods, the banding method of phosphorus application was better at each P level than a broadcasting method in both maize and wheat when values were averaged across the years and foliar applications. Foliar application of P solution increased the plant height at each increment of soil-applied P, with overall performance in the order of banding + foliar P > broadcast + foliar > banding with no foliar P > broadcast with no foliar P. Application of foliar P was better by 2.3% over no foliar, and banding was better by 1.4% over broadcast in maize and by 9.5 and 4.6% in wheat when averaged across the P levels and years. Similarly, the application of 20, 40 and 60 kg P ha^−1^ increased the maize plant heights by 5.4, 7.9 and 8.9%, respectively, and that of wheat plant heights by 6.1, 8.5 and 13.4%, respectively, when values were averaged across years, application methods and foliar sprays, indicating that the response of wheat was more pronounced than the maize crop.

### 2.2. Number of Grains (Maize and Wheat) Ear^−1^/Spike^−1^

The number of grains ear^−1^ in maize and grains spike^−1^ in wheat significantly improved with the foliar application when values were averaged across the P levels and methods of its application to soil (Table 2). The banding method of P application was better than broadcasting P fertilizers in both maize and wheat, whereas the year effect was non-significant for maize but wheat had a significantly higher number of grains spike in the second year as compared to the first year. Foliar application of 144 mM KH_2_PO_4_ at knee height + tasseling stages in maize increased the number of grains ear^−1^ at each increment of soil-applied P, irrespective of its application method to the soil. When averaged across the application methods, the foliar application of P increased the number of grains ear^−1^ from 338 to 357 and 366 to 413 at 0 and 20 kg soil-applied P ha^−1^, respectively, indicating its augmenting effect. Similarly, the wheat grains spike increased from 45 to 49 and 51 to 54 at given 0 and 20 kg P ha^−1^ soil-applied P levels, respectively. Furthermore, in both crops, the grains ear^−1^/spike^−1^ observed at lower P levels with foliar P was statistically at par or better than values observed at the next higher soil application level without foliar P, indicating supplementing soil-applied P and saving of fertilizer with foliar P application. The data showed that both banding- and broadcast-applied P supplemented with foliar P had higher grains ear^−1^ in maize and grains spike^−1^ in wheat when compared to sole application. The overall results showed that banding + foliar P was better than broadcast + foliar, whereas broadcast + foliar P was better than banding application alone, showing that the efficiency of the broadcasting method could be overcome through supplementing P with foliar application.

### 2.3. Thousand Grain Weight (g)

The number of grains ear^−1^/spike^−1^ and the 1000-grain weight of both maize and wheat increased with 144 mM KH_2_PO_4_ foliar sprays applied at knee height + tasseling in maize and stem elongation + boot stage in wheat during both years and at all soil P levels, irrespective of application method (Table 3). When averaged across P levels and years, 144 mM KH_2_PO_4_ foliar sprays increased the maize 1000-grain weight from 243 to 262 and wheat from 44 to 48 g, suggesting an improvement in the grain quality of both crops. Foliar application of 144 mM KH_2_PO_4_ increased the maize 1000-grain weight from 221 to 232 in control with 0 kg P ha^−1^ and from 263 to 282 g at 60 kg soil-applied P ha^−1^ when averaged across methods of soil application and years. Similarly, the wheat 1000-grain weight increased by 9.7, 7.0, 8.8 and 6.3% with the foliar application over no foliar plots at 0, 20, 40 and 60 kg P ha^−1^, respectively, when averaged across years and methods of soil application, suggesting a supplementing effect of foliar-applied P. Furthermore, the 1000-grain weight observed at any given soil P level with foliar P was either better or statistically at par to values obtained at the next higher soil P level for both crops, revealing fertilizer saving with foliar P. The same pattern has been depicted where both broadcast and banding supplemented with foliar P application showed increased 1000-grain weights of both maize and wheat. The overall results based on performance followed the pattern of banding + foliar > broadcast + foliar P > banding > broadcast at each level of soil-applied P. The results further indicated that broadcast + foliar P is better than banding without foliar P, which predicts that the lower P recovery associated with the broadcast method could be corrected with foliar P application.

### 2.4. Biological Yield (t ha^−1^)

Foliar application of 144 mM KH_2_PO_4_ solution, soil-applied P levels and method of application had significant (*p* ≤ 0.05) effects on the biological yield of maize and wheat during both years (Table 4). When values were averaged across the P levels for both maize and wheat, foliar application was better than non-foliar-applied plots and the banding method was statistically better than the broadcast method of soil P application. Foliar application of P increased the maize biological yield from 10.68 to 11.35 t ha^−1^ and that of wheat from 8.41 to 8.94 t ha^−1^ when averaged across the soil P levels, irrespective of application methods. Similarly, the banding method increased the maize yield from 10.88 to 11.14 t ha^−1^ and wheat from 8.54 to 8.81 t ha^−1^ when averaged across the soil P levels for both years, irrespective of foliar application. Application of foliar P at each increment of soil-applied P increased the biological yield of both maize and wheat. The biological yields of maize increased from 9 to 10.18 at 0 kg P ha^−1^ and similarly from 11.58 to 11.91 t ha^−1^ at 60 kg P ha^−1^, suggesting its supplementing effect at each increment of soil-applied P. The wheat yield increased by 7.25 to 8.22, 8.40 to 8.94, 8.88 to 9.20 and 9.10 to 9.40 kg P ha^−1^ with foliar P over no foliar-applied plots at 0, 20, 40 and 60 kg P ha^−1^, respectively. The yield obtained at any soil P levels + foliar P was better or statistically similar to the yields obtained with the next higher P level + no foliar P, indicating the supplementary effect of foliar P in saving of fertilizer and increasing P use efficiency. The overall results based on performance followed the pattern of banding + foliar P > broadcast + foliar P > banding alone > broadcast alone at any soil-applied P levels. Interestingly, the lower recovery associated with the broadcast method of P application could be overcome by foliar P application as broadcast + foliar P was better than banding + no foliar P at any soil-applied P level (Table 4).

### 2.5. Grain Yield

Foliar application of 144 mM KH_2_PO_4_ solution at knee height + tasseling stage of maize and stem elongation + boot stage of wheat significantly increased their grain yields at all soil-applied P levels, irrespective of its application method during both years (Table 5). When averaged across soil-applied P levels, application methods and years, the foliar P application at given growth stages increased maize grain yields from 4.17 to 4.48 kg ha^−1^ and those of wheat from 3.42 to 3.88 kg ha^−1^. The banding method was significantly better than the broadcast method in both crops when averaged across soil-applied P levels and years. Similarly, when averaged across application methods, years and foliar P, soil-applied P increased maize grain yields by 27.03, 36.33 and 39.24% and those of wheat by 27.7, 34.3 and 42.9% over the control with 20, 40 and 60 kg soil-applied P ha^−1^, respectively. The maize grain yields increased from 3.16 to 3.72 t ha^−1^ at soil 0 kg P ha^−1^ and from 4.71 to 4.88 t ha^−1^ at 60 kg P ha^−1^ with foliar P application when averaged across application methods, suggesting its supplementing effect at each soil-applied P level. Similar increases in wheat yields were 25, 10.5, 12.3 and 8.9% over no foliar-applied plots at 0, 20, 40 and 60 kg P ha^−1^, respectively. For both maize and wheat, the grain yield obtained at any soil-applied P level + foliar P was either better or statistically at par with the next higher soil-applied P level with no foliar P, suggesting its supplementing effect and saving of fertilizer with foliar P application. The same trend of saving of fertilizer with foliar P application was observed in both soil-applied methods, crops and years. The overall results based on effectiveness in increasing maize and wheat yields followed the pattern in the order of banding + foliar P > broadcast + foliar > banding + no foliar P > broadcast + no foliar P. Notably, the lower recovery and performance associated with broadcast method of soil P application could be corrected with foliar P as broadcast + foliar P produced higher maize and grain yields than banding + no foliar P at all soil P level. The grain yields of maize and wheat obtained at 40 kg P ha^−1^ + foliar P were better than yields obtained at 60 kg P ha^−1^ without foliar P, and hence, it seems a more suitable treatment for higher yield of maize and wheat in the prevailing soil, crop and climatic conditions of the area.

### 2.6. Harvest Index (%)

Foliar application of 144 mM KH_2_PO_4_ solution, soil-applied P levels and method of application had significant (*p* ≤ 0.05) effects on the harvest index of maize and wheat during both years (Table 6). When values were averaged across the P levels for both maize and wheat, foliar application was slightly better than non-foliar-applied plots. The banding method was statistically the same as the broadcast method of soil P application. The foliar application of P increased the harvest index in maize from 36 to 42%, whereas this increase was observed in wheat crop from 43 to 51% when combined with soil-applied P. The highest harvest index (HI) of 42% in maize and 51% in wheat was recorded at plots treated with 60 kg P ha^−1^, followed by 32.57% at plots treated with 40 kg ha^−1^, whereas the lowest were recorded 36 and 41% at control plots of maize and wheat, respectively.

### 2.7. Phosphorus Use Efficiency (PUE)

The P use efficiency, calculated as the increase in grain yield (kg ha^−1^) per unit application of foliar KH_2_PO_4_ (kg P ha^−1^) at given soil P levels applied either through a broadcast or banding method, showed a significant increase with foliar application 144 mM KH_2_PO_4_ (Table 7). When averaged across the application methods and years, the P use efficiency increased from 38.5 to 102.8 kg grain yield kg^−1^ P ha^−1^ in maize and from 32.5 to 111.7 kg grain yield kg^−1^ P ha^−1^ in wheat. The same increasing trends in PUE were observed at each increment of soil-applied P with such strength that PUE at any soil-applied P level with foliar P was higher than the PUE observed at the next higher P level without foliar P, suggesting its supplementing effect and saving of fertilizer. The PUE at lower soil-applied P levels was higher than PUE observed at higher soil P levels, affirming that the response of foliar P is more pronounced in P-deficient conditions, and as such, application of foliar P could correct midseason P deficiency. The PUE in the control was very high because in the control, there was no soil-applied P. The overall results showed that PUE followed a pattern in the order of banding + foliar P > broadcast + foliar P > banding + no foliar P > broadcast + no foliar P at each increment of soil-applied P, and the PUE decreased with increase in soil-applied P levels.

### 2.8. Nitrogen in Maize and Wheat Leaves

Maize and wheat leaf (N) at time of harvest on maturity significantly (*p* ≤ 0.05) varied with foliar P application, soil-applied P levels and method of application (Table 8). The increase in P levels to soil or plants increased the plant N, suggesting better assimilation of N and formation of amino acids and protein compounds with P supply to the plant. Phosphorus has been reported to remain involved in all metabolic activity, energy transfer and photosynthetic processes, hence its higher availability increases plant vigor, growth and conversion of photosynthate into proteins and other compounds. Foliar application of 144 mM KH_2_PO_4_ increased the maize and wheat leaf N at each increment of soil-applied P and at both application methods. The maize leaf (N) increased by 38.4, 50.3, 21.3 and 16.5% with foliar P at 0, 20, 40 and 60 kg P ha^−1^, respectively, whereas in wheat, such increases were 16.1, 22.4, 13.5 and 9.76% over the control, respectively, recording more response in rapidly growing maize than wheat. Similarly, the band application method of soil-applied P increased the maize leaf (N) from 1.80, 2.07 and 2.23 to 1.93, 2.19 and 2.34% at 20, 40 and 60 kg P ha^−1^, respectively, showing more increases with increase in expected P availability over broadcast application. In wheat, such increases were from 1.72, 1.98 and 2.08% to 1.86, 2.12 and 2.22% N at 20, 40 and 60 kg P ha^−1^, respectively. Notably, the application of foliar P at each increment of soil-applied P had higher maize and wheat leaf (N) than the next higher soil P level without foliar P, suggesting the supplanting effect and increase in P use efficiency with foliar P at each soil P level. Such increases in P use efficiency and leaf (N) resulted in higher maize and wheat yields in foliar-treated plots as compared to no foliar plots. The overall order based on performance followed the pattern of banding + foliar P > broadcast + foliar P > banding + no foliar P > broadcast + no foliar P. Foliar P has been reported to increase the plant leaf N if administered at the proper time.

### 2.9. Phosphorus in Maize and Wheat Leaves

The maize and wheat leaf (P) significantly increased with 144 mM KH_2_PO_4_ solution sprayed at the knee height + tasseling growth stage in maize and the stem elongation + boot stage in wheat, irrespective of soil-applied P level and method of application (Table 9). Foliar P application increased the maize leaf (P) from 0.17 to 0.22%, whereas in wheat, it varied from 0.13 to 0.16% when values were averaged across the soil P levels, methods of application and years. The banding method of P fertilizer application was significantly better in both maize and wheat in increasing leaf (P), which showed increases with increase in soil-applied P levels. The application of 20, 40 and 60 kg P ha^−1^ increased the maize leaf (P) by 18.8, 31.3 and 43.8% and that of wheat by 16.7, 25.0 and 33.3%, respectively, over controls, suggesting higher response in fast growing maize than in wheat. By comparing the various soil-applied treatments, it was noted that foliar application of 144 mM KH_2_PO_4_ increased the maize leaf (P) from 0.l4 to 0.19% at 0 kg P ha^−1^ and from 0.21 to 0.25% at 60 kg P ha^−1^, suggesting a supplementing effect at each soil-applied P level. Similar increases in wheat leaf (P) were by 18.18, 15.3, 21.42 and 11.76% at 0, 20, 40 and 60 kg P ha^−1^, respectively, affirming the increase in leaf (P) with foliar P. The same pattern of increase in maize and wheat leaf (P) with foliar application was observed in both methods of soil applications. Both maize and wheat leaf (P) increased with foliar P at each soil-applied P level, irrespective of application method, with an order of bandings + foliar P > broadcast + foliar P > banding + no foliar P > broadcast + no foliar P. The increase in maize and wheat leaf (P) with P fertilizer application is not uncommon, especially in low-fertility soils. Our results showed a linear increase in plant (P) with an increase in soil-applied P level that would be associated with a higher supply of P with an increase in application rate. Similarly, the increase from the banding method was significantly higher than from the broadcast method due to less interaction of soil-applied with P, leading to less P adsorption and making less complexes with soil cations in the banding method.

### 2.10. Potassium in Maize and Wheat Leaves

Application of 144 mM KH_2_PO_4_ also significantly increased the maize and wheat leaf (K) when sprayed at the knee height + tasseling stage in maize and the stem elongation + boot stage in wheat (Table 10). On the average basis with soil-applied P levels, the foliar P increased the maize leaf (K) from 3.24 to 3.58% and that of wheat leaf (K) from 3.2 to 3.6% over no foliar application. The maize and wheat leaf (K) also increased with soil-applied P levels as well as with its banding application, suggesting the P could improve the K assimilation in plants. When averaged across the application methods and years, there was a linear increase in leaf (K) with each increment of soil-applied P in both maize and wheat. In both maize and wheat, the leaf (K) observed at any soil P level with foliar P was higher than the next higher soil P level without foliar P, affirming its supplementing effect in increasing K assimilation and uptake. The increase in leaf (K) with foliar KH_2_PO_4_ could be associated directly as well as indirectly; i.e., KH_2_PO_4_ solution containing K as a component must have increased the leaf K due to its spraying on plant leaves (direct effect) and could be associated with a better supply of P to crops (indirect effect). The optimum supply of P increased the plant root growths, metabolic activity and plant vigor, which would have increased the leaf (K) as it did in the case of leaf (N). This phenomenon could be supported with the observed increase in leaf K with soil-applied P fertilizer as well as with the banding method of fertilizer application over the broadcast method. The results revealed that any methods that increased the availability and supply of P to plant also improved the plant leaf K.

### 2.11. Postharvest Soil NPK

The postharvest soil NPK contents, determined after harvest of wheat crop (both years), increased with foliar application of 144 mM KH_2_PO_4_ solution, soil-applied P levels and method of application. The observed increase in postharvest soil NPK could be direct, as with the application of P and K, as well as indirect, due to improvement in soil physical, biological and chemical properties associated with plant exudates, root growth and addition of plant litters. Foliar application of 144 mM KH_2_PO_4_ solution increased the soil total N from 0.92 to 1.16 g kg^−1^ (Table 11), P from 2.15 to 3.08 mg kg^−1^ (Table 12), and K from 113 to 134 mg kg^−1^ (Table 13) when values were averaged across years, soil P levels and method of application. Banding was a better method of application than broadcast, which increased the N, P and K from 1.04 to 1.05 g kg^−1^, 2.43 to 2.80 mg kg^−1^ and 119 to 127 mg kg^−1^, respectively. Similarly, soil-applied P at 20, 40 and 60 kg P ha^−1^ increased the soil N from 0.86 at control to 0.98, 1.12 and 1.19 g kg^−1^, P from 2.03 to 2.46, 2.83, 3.15 mg kg^−1^ and K from 106 to 120, 126 and 141 mg kg^−1^, respectively, showing an increase with each increment of soil-applied P. Conjunctive use of foliar-applied KH_2_PO_4_ supplemented the soil-applied P and further increased the postharvest soil NPK levels. For example, the soil N, P and K observed at 20 kg P ha^−1^ + foliar P were 1.07 g N kg^−1^, 2.97 mg P kg^−1^ and 133 mg K ha^−1^, which were not only improved over the given soil P but were also better than 0.98 g N kg^−1^, 2.35 mg P kg^−1^ and 113 mg K kg^−1^ observed at 40 kg P ha^−1^ without foliar P application. In our study, the increase in postharvest soil NPK is not dependent on an addition to the soil but rather the improvement associated with bumper growth of plants, especially for nitrogen and potassium.

## 3. Discussion

Phosphorus increases plant growth development by taking part in the metabolic activity, enhanced photosynthetic process and photosynthate assimilation [35], and, as such, increased plant height. Wahid et al. [36] reported that maize plant height increased with increase in P levels. Similarly, Singaram [37] observed rapid plant growth and development with the highest rate of P level. As with soil application of P, foliar application could also increase plant height by supplementing soil-applied P. The increases in plant height with foliar application are in agreement with Soylu et al. [38], Kenbaev and Sade [39] and Arif et al. [34], who reported a significant increase in plant height of wheat with foliar application of different NPK nutrients individually or in combination with each other.

The results showed that foliar P could augment the soil-applied P that may increase P use efficiency and reduce crop dependence on the soil. Dixon [17] suggested that foliar application of P could increase fertilizer use efficiency. The results obtained are in accordance with those of Sharma and Sharma [40] who reported that P fertilizer applications significantly increased the number of grains ear^−1^ in maize. The result was also similar to that of Maqsood et al. [41] who reported that numbers of grains ear^−1^ of maize were influenced significantly with NP foliar application. Arain et al. [42] reported that the number of grains ear^−1^ of maize increased with increase in P application. Similarly, Gooding and Devies [43] reported better performance of wheat for foliar application of N. Seth and Mosluh [44] also reported a marked increase in the number of grains per spike of wheat when urea was applied as a foliar spray.

Phosphorus being responsible for many enzymatic and metabolic activities and good root growth increased the thousand-grain weight and other growth and yielding parameters. The increases in 1000-grain weight were also in accordance with Fareed [45] and Hussain et al. [46], who observed an increase in 1000-grain weight with an increase in NP application. Soylu et al. [38] also reported a significant increase in thousand-grain weight with foliar application of nutrients. Similarly, Arif et al. [34] observed an increase in the thousand-grain weight of wheat with foliar application of NPK. Benbella and Paulsen [31] reported a similar increase in wheat yield with 2.2 and 4.4 kg P ha^−1^ sprayed at anthesis stages, but the application of 6.6 kg P ha^−1^ showed a decreasing trend and it was concluded that 2.2 kg ha^−1^ is the optimum rate for foliar P application. Mosali et al. [33] also reported that 2.0 kg P ha^−1^ was the best dose for higher yield of wheat applied at anthesis stage (Zadox-65) as a foliar spray.

These results are in line with those of Amanullah et al. [47] who reported that application of urea increased the number of leaves and plant height through foliar urea spray, which increased biological yield in maize by delaying the phenological development. Alston [48] also has reported that late-season foliar P application in wheat influenced biological yield. Pandey et al. [49] reported that foliar-applied P is absorbed by leaf stomata from which it is translocated to other parts of the plant and, after taking part in various biochemical reactions, ultimately increased the plant biomass. As compared to soil-applied P, foliar application of P in the prevailing alkaline conditions of the area could provide more benefits and chances of P absorption and assimilation by plants. Soleimani [50] reported an increase in biological yield of wheat by foliar application of phosphorus. Hamayun et al. [51] also observed the promising effect of foliar and soil application of NPK on the yield component of lentil. Our results also corroborate with the report of Arif et al. [34] who reported that foliar-applied P is more pronounced in P-deficient conditions as the response was more at rapid vegetative stages of the crop where the plant may suffer nutrient starvation because of the imbalance in supply and demand. Reuter et al. [52] and Poulsen et al. [53] also reported that foliar application of P increases wheat biological yield.

The increase in both maize and wheat yields with an increase in soil P levels revealed the response of P application in the given areas and crops. Marschner [54] concluded that P availability, which regulates photosynthesis and carbohydrate metabolism in the crop, was the most limiting factor during the reproductive stage. Arain et al. [42] also reported increases in grain yield of maize with an increase in P application. Our results were also in line with those of Hussain et al. [46], who reported that grain yield increased with phosphorus application, and plots receiving 40 kg P ha^−1^ gave the maximum grain yield as compared to the lower doses. Wheat crop needed 40 kg P ha^−1^ for maximum yields [55], showing a 43% increase over the control. The response of foliar P depends on the soil P level and soil having low P availability shows a higher response [56]. However, the response usually increased with an increase in foliar P concentration, up to a certain threshold level. Benbella and Paulsen [31] reported an at par increase in wheat yield with 2.2 and 4.4 kg P ha^−1^ sprayed at the anthesis stage, but application of 6.6 kg P ha^−1^ showed a decreasing trend, and it was concluded that 2.2 kg P ha^−1^ was the most suitable dose for foliar P application. They reported an increase in wheat yield by 1 t ha^−1^ with application of 1.1 to 2.2 kg P ha^−1^ (5 to 10 kg KH_2_PO_4_ ha^−1^).

The decrease in PUE with an increase in soil P level revealed that the response of foliar P was more pronounced in P-deficient conditions. Such results have been reported by Ali et al. [30] who observed the response to foliar P only when the plant P level was lower than the critical level of 0.3%.

## 4. Materials and Methods

### 4.1. Experimental Set Up

This study was conducted at a new developmental farm (NDF), the University of Agriculture, Peshawar, Pakistan, during the years 2014–2015. The physical and chemical characteristics of the soil prior to the experiment were determined in a composite soil sample as soil characteristics vary with land use [57,58,59]. The soil was silt loam in texture, alkaline (pH = 7.97), calcareous (Lime = 16.4%) and non-saline (0.43 dS m^−1^) in nature, low in organic matter (0.83%), available N (0.035%) and AB-DTPA available P content (2 mg kg^−1^). Series of field trials were conducted to evaluate the role of foliar P in supplementing the soil with applied P and in enhancing maize and wheat yields. Foliar P as 0 and 144 mM KH_2_PO_4_ [56] was sprayed on maize and wheat in addition to 0, 20, 40 and 60 kg P ha^−1^ applied [60] either through a broadcast or band method (5 cm away from seeds, 5 cm deep).

The treatments were arranged in three factorial randomized complete block designs (RCBD). The three factors were 2 types of foliar application (0 and 144 mM KH_2_PO_4_), two methods of soil P application (band and broadcast) and 4 levels of soil-applied P (0, 20, 40 and 60 kg P ha^−1^), making a total of 16 treatments replicated four times. Diammonium phosphate (DAP) (chemical formula (NH_4_)_2_HPO_4_) was used for soil-applied P, and potassium dihydrogen phosphate KH_2_PO_4_ for foliar supplementation was used. The soil application of P by both broadcast (BC) and banding (BD) methods was done at time of sowing as di-ammonium phosphate (DAP), whereas the foliar P as 144 mM KH_2_PO_4_ was sprayed at the knee-height (Zadocs-32)+ tasseling (Zadocs-65) stage to maize and at the stem elongation (Zadocs-22) + boot stage (Zadocs-45) to wheat in both growing seasons. Basal doses of N and K_2_O at the rate of 120 and 60 kg N and K_2_O were applied to all plots from urea and sulphate of potash (SOP) after correcting N levels from DAP. In wheat and maize, plot size was kept as 4 m × 5 m with an area 20 m^2^. In total, 48 treatment plots were made. The same layout was maintained for all four growing seasons (two maize and two wheat) and the same cultural practices, as per recommended procedures, were adopted. The maize cv. “Jalal” was grown on ridges with a seed rate of 30 kg ha^−1^ followed by wheat cv. “Atta Habib” with a seed rate of 120 kg ha^−1^ through hand drill during both years. The ridges in maize were spaced 70 cm from ridge to ridge, whereas in wheat, a 30-cm space was kept between rows. The wheat crop was harvested from the central 4 rows (length 5 m) at the ripping stage (Zadoks-92) and the maize crop was harvested about 20 days after the silk first appeared. Harvesting of wheat and maize was done manually by hand.

### 4.2. Agronomic Parameters

The data on plant height, number of grains ear^−1^/spike^−1^, 1000-grain weight, grain yield and biological yield were recorded along with postharvest and plant analysis in each treatment. For taking data on grain yield of maize, the ears were removed from the four central harvested rows and threshed with a small threshing machine, whereas in case of wheat, the harvested plants along with spikes were threshed by a small wheat threshing machine. In both cases, the grains were weighed on electronic balance and were converted to t ha with the following formula:

(1) $$\mathrm{Grain}\;\mathrm{yield}\;\left({t\;\mathrm{ha}}^{-1}\right)\;=\;\frac{\mathrm{weight}\;\left(\mathrm{kg}\right)\;}{4\;\mathrm{rows}}\;\times \frac{\mathrm{Total}\;\mathrm{rows}}{{\mathrm{Plot}\;\mathrm{size}\;m}^{2}}\;\times \frac{10000{\;m}^{2}}{\mathrm{ha}}\;\times \;\frac{\;t}{1000\;\mathrm{kg}}$$

### 4.3. Plant Analysis

The plant P was determined by the procedure of Kuo [61] after wet digestion in nitric acid and perchloric acid [62]. The same wet digest was also used for K analysis on a flame photometer (Jenway PF-7). The plant N and soil total N were determined by the procedure of Bremner [63]. AB-DTPA ext. P and K in soil were determined by the procedure of Soltanpour [64].

### 4.4. Soil Analysis

Organic matter in soil samples was determined by the Walkley–Black [65] procedure as described by Nelson and Sommers [66]. Soil pH was determined by the method determined by Thomas [67]. AB-DTPA extractable P was extracted in 1 N ammonium bicarbonate + 0.005 M diethylenetriamine pentaacetate (DTPA) solution suggested for alkaline calcareous soils [68], and P was determined through the NH_4_-molybdate complex method [61].

### 4.5. Statistical Analysis

The replicated data obtained were subjected to analysis of variance (ANOVA) according to two factorial randomized complete block design (RCBD) with split plot arrangement by using the procedure of Steel and Torrie [69] via the statistical package Statistix8.1 (Analytical Software, v 8.1, 1985–2005). The data were further subjected to the least significant difference (LSD) test for comparison of means where the results were significant.

## 5. Conclusions

Foliar application of 144 mM KH_2_PO_4_ administered at the knee height + tasseling stage in maize and the stem elongation + boot stage in wheat significantly increased their grain yields, growth and PUE at each soil P level, whether applied through banding or broadcast at the time of sowing. The overall increases in maize and wheat growth and yields at any soil P level followed a pattern in the order of banding + foliar P > broadcast + foliar P > banding + no foliar P > broadcast + no foliar P, indicating that the lower P response associated with the broadcast method could be corrected through foliar P application. The decreasing trends in response to an increase in soil P level proved an effective role of foliar P in P-deficient conditions and its higher suitability in calcareous soils, which usually suffer from severe P deficiency. Furthermore, the increase in PUE with foliar P was high enough to be considered economical and, hence, should be adopted for enhanced crop production in the area. However, on average, 60 kg P ha^−1^ is a more suitable soil application rate for wheat and maize. Keeping in view the promising effect of foliar P application in increasing PUE, this approach should be adopted and considered economical for improving crop production in alkaline calcareous soils. Furthermore, such studies should be conducted for other crops and locations for more widespread assessment, recommendation and adoption of the technology.

## Figures and Tables

**Table 1:** Maize and wheat plant heights (cm) as influenced by P solution at given soil-applied P levels in calcareous soils.

Foliar P	Methods	P Levels (kg ha^−1^)				Mean
		0	20	40	60	
**Maize**						
No Foliar spray	-	197	211	214	216	210 ^b^
Foliar P	-	205	213	219	221	215 ^a^
-	BC	200	209	215	218	211 ^b^
-	BD	202	215	219	219	214 ^a^
Year I	-	198	210	214	216	210 ^NS^
Year II	-	204	214	219	222	215
Mean		201 ^d^	212 ^c^	217 ^b^	219 ^a^	
**Wheat**						
No Foliar spray	-	78	82	86	90	84 ^b^
Foliar P	-	87	91	93	96	92 ^a^
-	BC	80	84	88	91	86 ^b^
-	BD	85	89	90	95	90 ^a^
Year I	-	82	85	88	92	87 ^NS^
Year II	-	83	88	90	94	89
Mean		82 ^d^	87 ^c^	89 ^b^	93 ^a^	

BC, BD and NS indicate broadcast application, band application and non-significant, respectively. Means with different letters in each column are significantly different (*p* ≤ 0.05).

**Table 2:** Maize and wheat grains ear^−1^/spike^−1^ as influenced by 144 mM KH_2_PO_4_ solution at given soil-applied P levels in calcareous soils.

Foliar P	Methods	P Levels (kg ha^−1^)				Mean
		0	20	40	60	
**Maize Crop**						
No Foliar	-	338	366	392	405	375 ^b^
Foliar P	-	357	413	422	436	407 ^a^
-	BC	343	379	394	416	383 ^b^
-	BD	352	400	421	426	399 ^a^
Year I	-	343	387	406	426	390
Year II	-	352	393	408	415	392
Mean		348 ^d^	390 ^c^	407 ^b^	421 ^a^	
**Wheat Crop**						
No Foliar	-	45	51	52	54	51 ^b^
Foliar P	-	49	54	56	58	54 ^a^
-	BC	46	51	53	54	51 ^b^
-	BD	48	54	55	57	53 ^a^
Year I	-	45	50	51	53	50
Year II	-	49	55	57	58	55
Mean		47 ^d^	52 ^c^	54 ^b^	56 ^a^	

BC and BD indicate broadcast application and band application, respectively. Means with different letters in each column are significantly different (*p* ≤ 0.05).

**Table 3:** Maize and wheat 1000-grain weights (g) as influenced by 144 mM KH_2_PO_4_ solution at given soil-applied P levels in calcareous soils.

Foliar P	Methods	P Levels (kg ha^−1^)				Mean
		0	20	40	60	
**Maize**						
No Foliar spray	-	221	242	244	263	243 ^b^
Foliar P	-	232	262	273	282	262 ^a^
-	BC	222	248	258	265	248 ^b^
-	BD	231	256	260	280	257 ^a^
Year I	-	220	244	254	268	246
Year II	-	232	260	264	278	258
Mean		226 ^d^	252 ^c^	259 ^b^	273 ^a^	
**Wheat**						
No Foliar spray	-	41	43	45	48	44 ^b^
Foliar P	-	45	46	49	51	48 ^a^
-	BC	43	44	46	47	45 ^b^
-	BD	43	45	48	51	47 ^a^
Year I	-	42	44	46	47	45
Year II	-	44	46	48	51	47
Mean		43 ^d^	45 ^c^	47 ^b^	49 ^a^	

BC and BD indicate broadcast application and band application, respectively. Means with different letters in each column are significantly different (*p* ≤ 0.05).

**Table 4:** Maize and wheat biological yield (t ha^−1^) as influenced by 144 mM KH_2_PO_4_ solution at given soil-applied P levels in calcareous soils.

Foliar P	Methods	P Levels (kg ha^−1^)				Mean
		0	20	40	60	
**Maize Crop**						
No Foliar	-	9.00	10.76	11.38	11.58	10.68 ^b^
Foliar P	-	10.18	11.52	11.78	11.91	11.35 ^a^
-	BC	9.56	10.86	11.44	11.66	10.88 ^b^
-	BD	9.62	11.41	11.72	11.83	11.14 ^a^
Year I	-	9.61	11.09	11.59	11.73	11.01 ^NS^
Year II	-	9.57	11.18	11.56	11.76	11.02
Mean		9.59 ^d^	11.14 ^c^	11.58 ^b^	11.75 ^a^	
**Wheat Crop**						
No Foliar	-	7.25	8.40	8.88	9.10	8.41 ^b^
Foliar P	-	8.22	8.94	9.20	9.40	8.94 ^a^
-	BC	7.67	8.49	8.85	9.15	8.54 ^b^
-	BD	7.80	8.86	9.24	9.35	8.81 ^a^
Year I	-	7.77	8.66	9.03	9.31	8.69 ^NS^
Year II	-	7.70	8.68	9.05	9.20	8.66
Mean		7.74 ^d^	8.67 ^c^	9.04 ^b^	9.25 ^a^	

BC, BD and NS indicate broadcast application, band application and non-significant, respectively. Means with different letters in each column are significantly different (*p* ≤ 0.05).

**Table 5:** Maize and wheat grain yield (t ha^−1^) as influenced by 144 mM KH_2_PO_4_ solution at given soil-applied P levels in calcareous soils.

Foliar P	Methods	P Levels (kg ha^−1^)				Mean
		0	20	40	60	
**Maize**						
No Foliar spray	-	3.16	4.24	4.57	4.71	4.17 ^b^
Foliar P	-	3.72	4.50	4.82	4.88	4.48 ^a^
-	BC	3.42	4.27	4.62	4.72	4.26 ^b^
-	BD	3.46	4.47	4.77	4.87	4.39 ^a^
Year I	-	3.42	4.38	4.67	4.75	4.31 ^NS^
Year II	-	3.47	4.36	4.71	4.84	4.34
Mean		3.44 ^d^	4.37 ^c^	4.69 ^b^	4.79 ^a^	
**Wheat**						
No Foliar spray	-	2.57	3.51	3.66	3.95	3.42 ^b^
Foliar P	-	3.21	3.88	4.11	4.30	3.88 ^a^
-	BC	2.90	3.55	3.76	4.04	3.56 ^b^
-	BD	2.88	3.83	4.01	4.22	3.73 ^a^
Year I	-	2.92	3.76	3.88	4.11	3.67 ^NS^
Year II	-	2.86	3.62	3.89	4.14	3.63
Mean		2.89 ^d^	3.69 ^c^	3.88 ^b^	4.13 ^a^	

BC, BD and NS indicate broadcast application, band application and non-significant, respectively. Means with different letters in each column are significantly different (*p* ≤ 0.05).

**Table 6:** Maize and harvest index (%) as influenced by 144 mM KH_2_PO_4_ solution at given soil-applied P levels in calcareous soils.

Foliar P	Methods	P Levels (kg ha^−1^)				Mean
		0	20	40	60	
**Maize**						
No Foliar spray	-	36	38	40	40	39 ^ab^
Foliar P	-	36	39	42	42	40 ^a^
-	BC	36	38	40	41	39 ^a^
-	BD	36	39	41	41	39 ^a^
Year I	-	37	40	41	41	40
Year II	-	35	38	41	41	39
Mean		36 ^d^	39 ^c^	41 ^ab^	41^a^	
**Wheat**						
No Foliar spray	-	41	42	42	46	43 ^b^
Foliar P	-	43	45	47	51	47 ^a^
-	BC	42	43	43	47	44 ^b^
-	BD	43	45	47	50	46 ^a^
Year I	-	42	44	44	49	45
Year II	-	43	43	46	48	45
Mean		42 ^d^	44 ^c^	45 ^b^	49 ^a^	

BC and BD indicate broadcast application and band application, respectively. Means with different letters in each column are significantly different (*p* ≤ 0.05).

**Table 7:** Phosphorus use efficiency (kg grain yield kg^−1^ P ha^−1^) of maize and wheat as influenced by 144 mM KH_2_PO_4_ solution at given soil-applied P levels in calcareous soils.

Foliar P	Methods	P Levels (kg ha^−1^)				Mean
		0	20	40	60	
**Maize**						
No Foliar spray	-	-	54.3	35.3	25.9	38.5
Foliar P	-	283.2	61.0	39.5	27.7	102.8
-	BC	-	53.3	35.8	25.8	38.3
-	BD	-	62.0	39.0	27.8	42.9
Year I	-	277.6	59.0	37.4	26.4	100.1
Year II	-	288.8	56.3	37.4	27.2	102.4
Mean		283.2	57.6	37.4	26.8	
**Wheat**						
No Foliar spray	-	-	47.0	27.3	23.1	32.5
Foliar P	-	322.5	59.7	36.7	27.9	111.7
-	BC	-	43.1	27.3	22.9	31.1
-	BD	-	63.6	36.7	28.2	42.8
Year I	-	336.5	55.8	31.5	25.0	112.2
Year II	-	308.5	50.9	32.6	26.1	104.5
Mean		322.5	53.3	32.0	25.5	

BC and BD indicate broadcast application and band application, respectively.

**Table 8:** Maize and wheat grain leaf nitrogen (%) at maturity as influenced by 144 mM KH_2_PO_4_ solution at given soil-applied P levels in calcareous soils.

Foliar P	Methods	P Levels (kg ha^−1^)				Mean
		0	20	40	60	
**Maize**						
No Foliar spray	-	1.38	1.49	1.92	2.11	1.73 ^b^
Foliar P	-	1.91	2.24	2.33	2.46	2.23 ^a^
-	BC	1.58	1.80	2.07	2.23	1.92 ^b^
-	BD	1.71	1.93	2.19	2.34	2.04 ^a^
Year I	-	1.68	1.83	2.04	2.22	1.94
Year II	-	1.60	1.90	2.21	2.35	2.02
Mean		1.64 ^d^	1.87 ^c^	2.13 ^b^	2.28 ^a^	
**Wheat**						
No Foliar spray	-	1.43	1.61	1.92	2.05	1.75 ^b^
Foliar P	-	1.65	1.97	2.18	2.25	2.01 ^a^
-	BC	1.49	1.72	1.98	2.08	1.82 ^b^
-	BD	1.59	1.86	2.12	2.22	1.95 ^a^
Year I	-	1.58	1.79	2.05	2.14	1.89
Year II	-	1.49	1.79	2.05	2.16	1.87
Mean		1.54 ^d^	1.79 ^c^	2.05 ^b^	2.15 ^a^	

BC and BD indicate broadcast application and band application, respectively. Means with different letters in each column are significantly different (*p* ≤ 0.05).

**Table 9:** Maize and wheat grain leaf phosphorus (%) at maturity as influenced by 144 mM KH_2_PO_4_ solution at given soil-applied P levels in calcareous soils.

Foliar P	Methods	P Levels (kg ha^−1^)				Mean
		0	20	40	60	
**Maize**						
No Foliar spray	-	0.14	0.15	0.19	0.21	0.17 ^b^
Foliar P	-	0.19	0.22	0.23	0.25	0.22 ^a^
-	BC	0.16	0.18	0.21	0.22	0.19 ^b^
-	BD	0.17	0.19	0.22	0.23	0.21 ^a^
Year I	-	0.17	0.18	0.20	0.22	0.19
Year II	-	0.16	0.19	0.22	0.24	0.20
Mean		0.16 ^d^	0.19 ^c^	0.21 ^b^	0.23 ^a^	
**Wheat**						
No Foliar spray	-	0.11	0.13	0.14	0.15	0.13 ^b^
Foliar P	-	0.13	0.15	0.17	0.17	0.16 ^a^
-	BC	0.12	0.13	0.15	0.15	0.14 ^b^
-	BD	0.13	0.14	0.16	0.17	0.15 ^a^
Year I	-	0.12	0.14	0.15	0.16	0.14
Year II	-	0.12	0.14	0.15	0.16	0.14
Mean		0.12 ^d^	0.14 ^c^	0.15 ^b^	0.16 ^a^	

BC and BD indicate broadcast application and band application, respectively. Means with different letters in each column are significantly different (*p* ≤ 0.05).

**Table 10:** Maize and wheat grain leaf potassium (%) at maturity as influenced by 144 mM KH_2_PO_4_ solution at given soil-applied P levels in calcareous soils.

Foliar P	Methods	P Levels (kg ha^−1^)				Mean
		0	20	40	60	
**Maize**						
No Foliar spray	-	2.77	3.09	3.42	3.68	3.24 ^b^
Foliar P	-	3.02	3.52	3.80	3.98	3.58 ^a^
-	BC	2.80	3.26	3.56	3.81	3.36 ^b^
-	BD	2.99	3.34	3.67	3.85	3.46 ^a^
Year I	-	2.71	3.22	3.47	3.69	3.27
Year II	-	3.07	3.38	3.76	3.96	3.54
Mean		2.89 ^d^	3.30 ^c^	3.61 ^b^	3.83 ^a^	13.64
**Wheat**						
No Foliar spray	-	2.7	3.2	3.4	3.5	3.2 ^b^
Foliar P	-	3.2	3.5	3.8	3.9	3.6 ^a^
-	BC	2.9	3.3	3.5	3.7	3.3 ^b^
-	BD	3.1	3.4	3.7	3.8	3.5 ^a^
Year I	-	2.9	3.3	3.5	3.7	3.4
Year II	-	3.1	3.4	3.6	3.8	3.5
Mean		3.0 ^d^	3.4 ^c^	3.6 ^b^	3.7 ^a^	

BC and BD indicate broadcast application, band application, respectively. Means with different letters in each column are significantly different (*p* ≤ 0.05).

**Table 11:** Postharvest soil total N (g kg^−1^) as influenced by maize and wheat cropping supplied with 144 mM KH_2_PO_4_ foliar solution at given soil-applied P levels in calcareous soils.

Foliar P	Method	P Levels (kg ha^−1^)				Mean
		0	20	40	60	
**Year I**						
0	BC	0.70	0.89	1.01	1.05	0.9
0	BD	0.75	0.90	0.93	1.03	0.9
144 mM	BC	0.85	1.02	1.25	1.35	1.1
144 mM	BD	0.93	1.09	1.31	1.40	1.2
**Year II**						
0	BC	0.88	0.95	1.03	1.09	1.0
0	BD	0.81	0.85	0.95	0.89	0.9
144 mM	BC	0.96	1.08	1.23	1.37	1.2
144 mM	BD	0.98	1.11	1.28	1.36	1.2
**Average Across Methods**						
No Foliar spray	-	0.79	0.89	0.98	1.02	0.92 ^b^
Foliar P	-	0.93	1.07	1.27	1.37	1.16 ^a^
-	BC	0.85	0.98	1.13	1.22	1.04 ^a^
-	BD	0.87	0.99	1.12	1.17	1.04 ^a^
Year I	-	0.81	0.97	1.12	1.21	1.03
Year II	-	0.91	1.00	1.12	1.18	1.05
Mean		0.86 ^d^	0.98 ^c^	1.12 ^b^	1.19 ^a^	

BC and BD indicate broadcast application, band application, respectively. Means with different letters in each column are significantly different (*p* = 0.05).

**Table 12:** Postharvest soil ammonium bicarbonate-diethylenetriamine pentaacetate (AB-DTPA) extrated P (mg kg^−1^) as influenced by maize and wheat cropping supplied with 144 mM KH_2_PO_4_ foliar solution at given soil-applied P levels in calcareous soils.

Foliar P	Method	P Levels (kg ha^−1^)				Mean
		0	20	40	60	
**Year I**						
0	BC	1.37	1.65	2.13	2.45	1.90
0	BD	1.72	2.07	2.40	2.81	2.25
144 mM	BC	2.24	2.74	3.02	3.33	2.83
144 mM	BD	2.55	3.02	3.41	3.73	3.17
**Year II**						
0	BC	1.49	1.81	2.33	2.60	2.05
0	BD	1.80	2.26	2.57	3.01	2.41
144 mM	BC	2.35	2.81	3.20	3.42	2.94
144 mM	BD	2.74	3.34	3.57	3.86	3.38
**Average Across Methods**						
No Foliar spray	-	1.59	1.94	2.35	2.72	2.15 ^b^
Foliar P	-	2.47	2.97	3.30	3.58	3.08 ^a^
-	BC	1.86	2.25	2.67	2.95	2.43 ^b^
-	BD	2.20	2.67	2.99	3.35	2.80 ^a^
Year I	-	1.97	2.37	2.74	3.08	2.54
Year II	-	2.09	2.55	2.92	3.22	2.70
Mean		2.03 ^d^	2.46 ^c^	2.83 ^b^	3.15 ^a^	

BC and BD indicate broadcast application and band application, respectively. Means with different letters in each column are significantly different (*p* = 0.05).

**Table 13:** Postharvest soil AB-DTPA ext. K (mg kg^−1^) as influenced by maize and wheat supplied with 144 mM KH_2_PO_4_ foliar solution at given soil-applied P levels in calcareous soils.

Foliar P	Method	P Levels (kg ha^−1^)				Mean
		0	20	40	60	
**Year I**						
0	BC	89	96	105	134	105.8
0	BD	99	109	120	132	114.9
144 mM	BC	111	127	134	138	127.3
144 mM	BD	122	135	140	143	134.8
**Year II**						
0	BC	92	106	110	140	111.8
0	BD	105	113	117	138	118.1
144 mM	BC	110	131	141	149	132.5
144 mM	BD	124	140	145	153	140.3
**Average Across Methods**						
No Foliar spray	-	96	106	113	136	113 ^b^
Foliar P	-	117	133	140	146	134 ^a^
-	BC	100	115	122	140	119 ^b^
-	BD	112	124	130	141	127 ^a^
Year I	-	105	117	124	137	121
Year II	-	108	122	128	145	126
Mean		106 ^d^	120 ^c^	126 ^b^	141 ^a^	

BC and BD indicate broadcast application and band application, respectively. Means with different letters in each column are significantly different (*p* = 0.05).
